# Light transmittance of zirconia as a function of thickness and microhardness of resin cements under different thicknesses of zirconia

**DOI:** 10.4317/medoral.18181

**Published:** 2013-02-05

**Authors:** Isil Cekic-Nagas, Ferhan Egilmez, Gulfem Ergun, Bekir M. Kaya

**Affiliations:** 1 DDS, PhD, Research Associate, Gazi University, Faculty of Dentistry, Department of Prosthodontics, Ankara, Turkey; 2 DDS, PhD, Associate Professor, Gazi University, Faculty of Dentistry, Department of Prosthodontics, Ankara, Turkey; 3 DDS, Research Assistant, Gazi University, Faculty of Dentistry, Department of Prosthodontics, Ankara, Turkey

## Abstract

Objective: The objective of this study was to compare microhardness of resin cements under different thicknesses of zirconia and the light transmittance of zirconia as a function of thickness. Study design: A total of 126 disc-shaped specimens (2 mm in height and 5 mm in diameter) were prepared from dual-cured resin cements (RelyX Unicem, Panavia F and Clearfil SA cement). Photoactivation was performed by using quartz tungsten halogen and light emitting diode light curing units under different thicknesses of zirconia. Then the specimens (n=7/per group) were stored in dry conditions in total dark at 37°C for 24 h. The Vicker’s hardness test was performed on the resin cement layer with a microhardness tester. Statistical significance was determined using multifactorial analysis of variance (ANOVA) (alpha=.05). Light transmittance of different thicknesses of zirconia (0.3, 0.5 and 0.8 mm) was measured using a hand-held radiometer (Demetron, Kerr). Data were analyzed using one-way ANOVA test (alpha=.05). Results: ANOVA revealed that resin cement and light curing unit had significant effects on microhardness (p < 0.001). Additionally, greater zirconia thickness resulted in lower transmittance. There was no correlation between the amount of light transmitted and microhardness of dual-cured resin cements (r = 0.073, p = 0.295). Conclusion: Although different zirconia thicknesses might result in insufficient light transmission, dual-cured resin cements under zirconia restorations could have adequate microhardness.

** Key words:**Zirconia, microhardness, light transmittance, resin cement.

## Introduction

The optical property of high strength ceramic materials such as glass infiltrated spinell and alumina or fused alumina or zirconia core materials plays an important role in matching the affected shade of the artificial restorations with the natural appearance of teeth (1,2). Additionally, core translucency is one of the primary factors in controlling esthetics and a critical consideration in the selection of the material (3,4). Translucency is largely dependent on light scattering. When the majority of light beam, in passing through a material, is partly scattered and diffusely reflected, the material will appear opaque. If only a part of the light is scattered and the most is diffusely transmitted, the material will appear translucent. Moreover, the translucency of the material depends on the amount of crystals within the core matrix, their chemical nature, and the size of the particles (5,6).

Traditional luting agents (such as zinc-phosphate or resin-modified glass ionomer cements) might provide adequate retention for cementation of zirconium oxide restorations. However, adhesive cementation with adhesive luting agents is necessary for ensuring better retention and marginal adaptation (7).

Until recently, conventional quartz tungsten halogen (QTH) light curing units (LCUs) were widely used to polymerize resin cements. In the last few years, other technologies, such as xenon plasma arc (PAC) and light-emitting diodes (LED) are also available (2). Although these systems are still being developed, the effectiveness of these LCUs has been recently questioned.

The polymerization of resin cements is critical, because inadequate polymerization is usually associated with poor mechanical and biological properties of the resin cements. Furthermore, modified light polymerization protocols can lead to the resulting polymer having different structures and properties, such as hardness, tensile strength, and water solubility and sorption. Surface hardness is one of the most important physical properties of dental materials, and is defined as the resistance of a material to indentation or penetration. Hardness testing is commonly used as a simple and reliable method to indicate the degree of conversion of resin-based cements (2,8).

Based on these considerations, the first aim of this study was to compare the microhardness of resin cements polymerized with different LCUs under different thicknesses of zirconia. Additionally, the second aim was to evaluate the translucency of zirconia as a function of the thickness. The tested null hypothesis was twofold: 1- Polymerization of resin cements with different LCUs significantly affects the microhardness values. 2- Light transmittance of zirconia and as a result microhardness of resin cements under zirconia decrease when the thickness of zirconia is increased.

## Material and Methods

**Figure 1:**
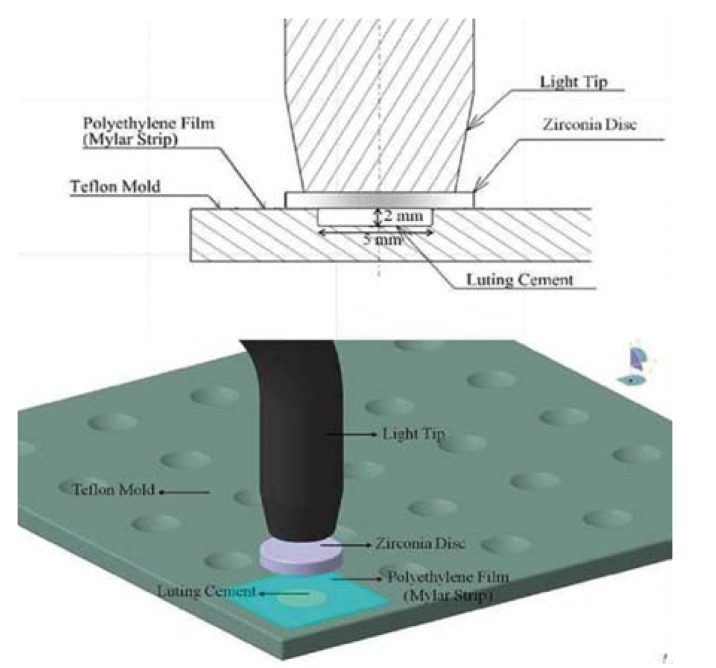
Schematic illustration of specimen preparation.

The experimental set-up is given in figure 1.

The tested resin cements (RelyX Unicem, Panavia F and Clearfil SA cement) their manufacturers and compositions were shown in  Table 1. A total of 126 disc-shaped specimens were prepared by the same operator from different resin cements (RelyX Unicem, Panavia F and Clearfil SA cement) according to the manufacturers’ directions (n=42/per group). Each disk was 5 mm in diameter and 2±0.01 mm thickness. Pastes A and B of the Panavia F hand-mixing cement was mixed in a 1:1 ratio on a mixing pad for 10 s. The other self-mixing cements (Clearfil SA cement and RelyX Unicem) were mixed by activating the syringes and triturating for 5 s. After placing the resin cements in a circular polytetrafluoroethylene mold, a transparent polyethylene film was placed over the filled mold and the zirconia discs (10 mm in diameter) in different thicknesses (0.3, 0.5 and 0.8 mm) were positioned over the resin cement (n=14/per group). The specimens were provided as 2 groups (n=7/per group) according to the LCU used (polymerized for 20 s): a quartz tungsten halogen (QTH, Blue Swan Digital; Dentanet, Turkey) and a light-emitting diode (LED, Elipar Freelight 2; 3M Espe, St. Paul, MN, USA). Then the specimens were stored dry in dark at 37°C for 24 h.

**Table 1:**
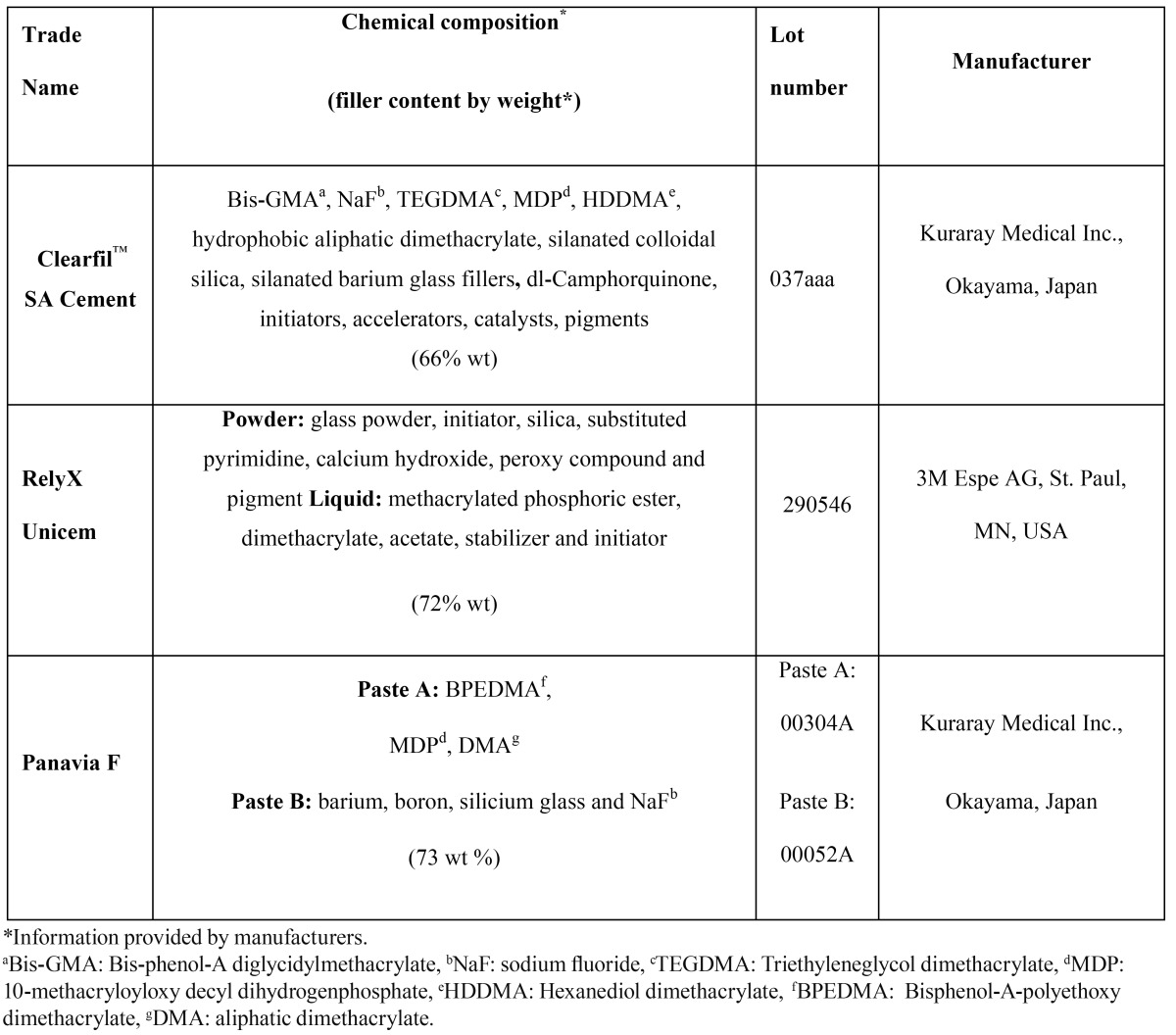
Test materials and their composition according to manufacturers.

**Figure 2:**
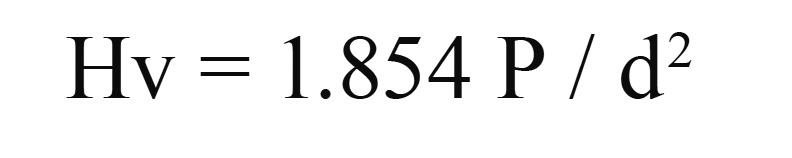
Calculation of VHN.

The specimens were polished with 220, 360, and 600 grit silicone carbide abrasive paper (Federation of European Producers of Abrasives (FEPA) under water-cooling with a grinding machine (Struers RotoPol 11, Struers A/S, Rodovre, Denmark) and placed on the platform of the tester with the surface being tested facing the diamond indenter. The Vicker’s hardness number (VHN) test was performed on the cement layer with a microhardness tester (Shimadzu HMV; Shimadzu Corporation, Tokyo, Japan) with 200 g of load application for 15 seconds. Three indentations were taken for each of the top and the bottom surfaces of each resin cement specimen. These indentations were not closer than 1 mm to the margin and were averaged to determine the hardness value for each specimen. VHN was calculated using the following formula: (Fig. 2).

where P is the load in kilograms and d is the diagonal length of the indent in millimeters.

Translucency measurement:

Zirconia (Whitepeaks Dental Systems Co, Essen, Germany) disc-shaped specimens (n=24) (in A1 shade) were fabricated by the manufacturer using a computer aided design/computer-aided milling (CAD/CAM) process in three different thicknesses (0.3, 0.5 and 0.8 mm) (n=8/per group). Before measurement of translucency, the zirconia discs were ultrasonically cleaned in distilled water for 10 minutes and then dried with compressed air. The thickness of the discs was measured using a digital micrometer (Mitutoyo Manufacturing Company Ltd, Kawasaki, Japan). The accuracy of the micrometer was ±10μm.

The light transmission value of each thickness was measured by placing the disc on the aperture of the hand-held radiometer (Demetron, Kerr, Orange, CA, USA) and recording the average of resultant light readings through the disc. Then transmittance percentage was calculated for each thickness.

-Statistical analysis

Multifactorial analysis of variance (ANOVA) was performed to determine the effects of different resin cements, LCUs and zirconia thicknesses on the Vicker’s microhardness of resin cements. Paired-Samples T test was used to compare the microhardness values of resin cements in top and bottom surfaces. A p value less than 0.05 was considered statistically significant. But, for all possible multiple comparison tests, Bonferroni Adjustment was applied to control Type I error. The transmittance data were analyzed using one-way Anova and Tukey’s post-hoc tests (alpha=.05). A non-parametric Spearman’s correlation analysis and Spearman’s coefficient (rho) was used to assess the correlation between the microhardness of resin cements and light transmission through zirconia ceramics.

## Results

The mean microhardness values and standard deviations for specimens are shown in  Table 2. Statistical analysis demonstrated that resin cement and light curing unit had significant effects on microhardness (p < 0.001). Furthermore, thickness of zirconia had no significant effect on microhardness of resin cements in both surfaces (top and bottom surfaces) (p = 0.151 and p = 0.088, respectively). A significant two factor interaction between resin cement and LCUs was observed in both surfaces (p < 0.001). However, no three factor interaction was observed in both surfaces among resin cements, LCUs and different thicknesses of zirconia (top surface: p = 0.932 and bottom surface: p = 0.517).

**Table 2:**
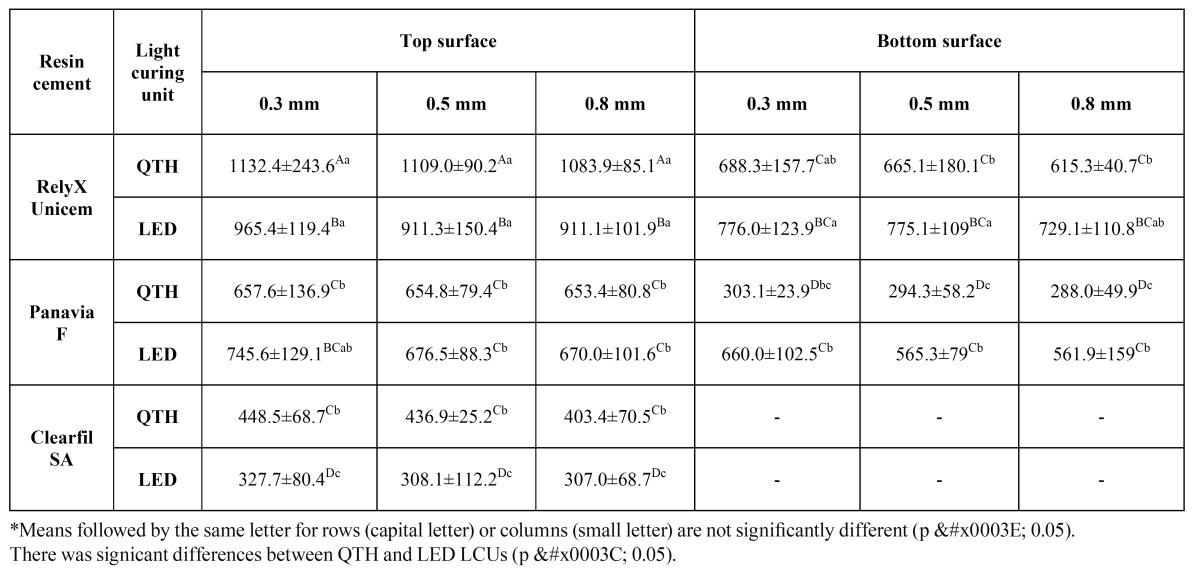
The mean microhardness and standard deviation values of the resin cements tested.

The results of the microhardness test indicated significant differences based on the surface of resin cements (top and bottom surface) (p < 0.004). The top surface microhardness values of Rely X Unicem and Clearfil SA cement when polymerized with QTH LCU were higher than the results when polymerized with LED LCU (p < 0.001). Moreover, the bottom surface microhardness values of Rely X Unicem and Panavia F when polymerized with QTH LCU were lower than the results when polymerized with LED LCU (p < 0.001). However, the microhardness of Clearfil SA cement wasn’t detectable on the bottom surface. The impression of the indenter couldn’t be clearly identified on the surface of the Clearfil SA cement specimens polymerized under three different thicknesses of zirconia discs. In addition to this, the top surface microhardness values of Panavia F when polymerized with QTH LCU were similar to the results when polymerized with LED LCU (p = 0.102).

The microhardness of the tested materials in the upper surface can be rank as follows: Rely X Unicem > Panavia F > Clearfil SA cement (p < 0.001). Furthermore, statistically significant ranking for microhardness was obtained in the bottom surface as follows: Rely X Unicem > Panavia F (p < 0.001).

**Figure 3:**
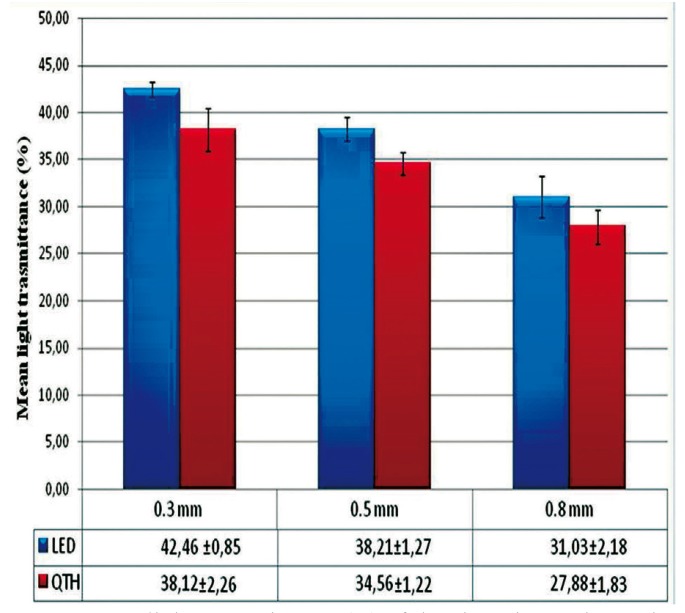
Mean light transmittance (%) of the zirconia specimens in different thicknesses.

Transmittance percentage values of various zirconia discs are displayed in figure 3. One-way ANOVA revealed the factor of “thickness” in this study, demonstrated the statistically significant effect on light transmittance (p < 0.001). The percentage of light trasmittance was ranked as follows: 0.3 mm > 0.5 mm > 0.8 mm (p < 0.001). As a result, percentage of light transmittance was decreased with the increase in thickness of zirconia disc.

## Discussion

It is known that to reach optimum physical properties of resin cements under ceramic restorations; degree of conversion should be as high as possible (9). In the present study, to simulate clinical conditions, the resin cement specimens were irradiated from the top of the zirconia discs using LCUs, where the end of the light guide was in contact with the discs.

The first null hypothesis was accepted after statistical analysis revealed differences in microhardness among the resin cements after light curing with different LCUs. The hardening of resin cements was investigated to ensure the efficacy of different LCUs. Previous studies showed hardness as a good indicator of conversion of double bonds (10,11) and was therefore used in the present study as an indirect measurement of degree of conversion. It was also reported that hardness was useful in determining the development of the mechanical properties of resin composites during their polymerization reaction, and that there was a direct correlation between degree of conversion and hardness development during polymerization, as a consequence of the increase in stiffness and strength of the material (12,13).

Conflicting results are often indicated in the literature when the effects of different LCUs on resin based materials are reported (14,15). Previous studies reported mechanical properties of LED LCU polymerized resin composites as well or better than some QTH LCUs (16,17). A previous study by Cekic-Nagas et al. (2) compared the polymerization ability of three different light-curing units (QTH, LED and PAC) by determination of microhardness and found significant effects of resin cement and LCU on microhardness. Similar to these previous studies, in the present study, LED LCU presented higher or similar hardness values than QTH on the top surface of the resin cements. However, on the bottom surface of Panavia F and Rely X Unicem, LED LCU presented lower hardness values than QTH.

A previous study by Aguiar et al. (18) evaluated the influence of light curing modes and curing time on the microhardness of a hybrid composite resin and indicated higher hardness values of top surfaces than bottom surfaces. In accordance to that previous study, top surfaces of resin cements showed higher microhardness values than bottom concerning LCUs, resin cements and zirconia thickness ( Table 2). This finding might be indicative of the limitation of transmitted light passing through 2 mm thickness of resin cements.

Further on the comparison of microhardness values of the resin cements, Rely X Unicem showed higher microhardness values than Panavia F and Clearfil SA cements ( Table 2). This could be partially attributed to the differences in filler load, filler type, resin matrix, and formulation. The filler particles incorporated into the matrix provide much better mechanical properties than the matrix itself. Therefore, up to a certain limit, a higher filler load may be expected to improve mechanical properties. A previous study by Pilo and Cardash demonstrated a correlation between filler content and hardness (19). Differences between the resin-based cements included in the present study, can be explained by their different filler content. The amounts of fillers used in this study were 72% and 73% by weight for Rely X Unicem and Panavia F respectively, which were higher than Clearfil SA cement (66%) ( Table 1). Rely X Unicem, having high filler load also ranked highest surface hardness. Clearfil SA, having lower filler load, in their turn showed a lower hardness. However, in the current study, Panavia F, with highest filler content (73%), had lower values than Rely X Unicem. The reason for this could be that the use of hand-mixing Panavia F, unlike the other self-mixing cements tested in this study, might have resulted in mixing errors associated with different polymerization initiators and reactions. Self-mixing may help discard susceptibility to operator-induced variability (20). Thus, lower hardness results of Panavia F could be related with the air inclusions during the mechanical mixing or consistency of the material, which might more readily accept air inclusion during the hand-mixing process (2). Moreover, Clearfil SA, consisting TEGDMA, presented lower values for hardness. In accordance with the present study, a recent study by Moraes et al. (21) confirmed that hardness is lower for TEGDMA-rich resin cements. Furthermore, in the evaluation process of Clearfil SA, the microhardness values of this cement couldn’t be obtained on the bottom surface. The inside of the Clearfil SA cement specimens, there were many defects and cracking lines; when a diamond tip of the hardness tester was pressed on these defects and cracking lines, microhardness levels were evidently decreased, even couldn’t be measured. This might suggest an inadequate polymerization of Clearfil SA cement on the bottom surface.

Knowledge of the optical properties of dental restorative materials is very important to achieve esthetic restorations, and this study attempts to analyze optical properties of the zirconia ceramics. The second null hypothesis that the light transmittance of zirconia decreases when the thickness is increased should be partially accepted, as the different thicknesses of zirconia had a significant effect on light transmittance values of the tested zirconia specimens. This finding is according to the results of a previous study by Lee et al. (22), suggesting a decrease in light transmittance with an increase in thickness of porcelain disc. Similarly, Antonson et al. (23) indicated that translucency is inversely related to the thickness of the ceramic layer to be traversed by the light beam and is strongly influenced by light scattering.

Previous studies have reported an inverse relationship between the thickness of ceramic inlays and the surface hardness of resin cements (24,25). However, the results of this study demonstrated that polymerization of dual-cured resin cements under different zirconia thicknesses had no effect on microhardness. A previous study by Kilinc et al. (25) evaluated the effect of ceramic thickness and shade on the microhardness of various light-cured and dual-cured resin cements and concluded that the polymerization was adversely affected only when the ceramic thickness was 3 mm and above. The zirconia thicknesses investigated in the present study were less than 3 mm. That could be the reason for similar microhardness values of the resin cements under different zirconia thicknesses. In the context of this study, no correlation was found between light transmission through zirconia and microhardness of resin cements. At this juncture, it should be put into perspective that, dual-cured resin cements were tested in the present study and the transmitted light had no effect on microhardness of these cements. This may be related with the ongoing chemical polymerization of dual-cured resin cements after light polymerization.

## Conclusion

As microhardness of the resin based material might give an idea about the polymerization process, further investigations are required to test the microhardness of resin cements in vitro and in vivo. Additionally, further investigations about optical properties should be studied before and after the application of veneering porcelain on zirconia core materials. Within the limitations of this in-vitro study, the following conclusions could be drawn:

1. High-power LED LCUs might be considered as effective as QTH LCU or more effective than QTH LCU for polymerization of the resin-based materials.

2. Greater thickness of zirconia results in lower light transmittance.

3. There might be insufficient light transmission through zirconia restorations for adequate microhardness. Thus, use of dual-cured resin cements might be preferred under zirconia restorations.

## References

[1] Shiraishi T, Wood DJ, Shinozaki N, van Noort R (2011). Optical properties of base dentin ceramics for all-ceramic restorations. Dent Mater.

[2] Cekic-Nagas I, Ergun G (2011). Effect of different light curing methods on mechanical and physical properties of resin-cements polymerized through ceramic discs. J Appl Oral Sci.

[3] Jiang L, Liao Y, Wan Q, Li W (2011). Effects of sintering temperature and particle size on the translucency of zirconium dioxide dental ceramic. J Mater Sci Mater Med.

[4] Spyropoulou PE, Giroux EC, Razzoog ME, Duff RE (2011). Translucency of shaded zirconia core material. J Prosthet Dent.

[5] Heffernan MJ, Aquilino SA, Diaz-Arnold AM, Haselton DR, Stanford CM, Vargas MA (2002). Relative translucency of six all-ceramic systems. Part I: core materials. J Prosthet Dent.

[6] Baldissara P, Llukacej A, Ciocca L, Valandro FL, Scotti R (2010). Translucency of zirconia copings made with different CAD/CAM systems. J Prosthet Dent.

[7] de Oyagüe RC, Monticelli F, Toledano M, Osorio E, Ferrari M, Osorio R (2009). Influence of surface treatments and resin cement selection on bonding to densely-sintered zirconium-oxide ceramic. Dent Mater.

[8] Aguiar FH, Braceiro AT, Ambrosano GM, Lovadino JR (2005). Hardness and diametrical tensile strength of a hybrid composite resin polymerized with different modes and immersed in ethanol or distilled water medium. Dent Mater.

[9] Jung H, Friedl KH, Hiller KA, Haller A, Schmalz G (2001). Curing efficiency of different polymerization methods through ceramic restorations. Clin Oral Investig.

[10] David JR, Gomes OM, Gomes JC, Loguercio AD, Reis A (2007). Effect of exposure time on curing efficiency of polymerizing units equipped with light-emitting diodes. J Oral Sci.

[11] Bouschlicher MR, Rueggeberg FA, Wilson BM (2004). Correlation of bottom-to-top surface microhardness and conversion ratios for a variety of resin composite compositions. Oper Dent.

[12] Reges RV, Moraes RR, Correr AB, Sinhoreti MA, Correr-Sobrinho L, Piva E (2008). In-depth polymerization of dual-cured resin cement assessed by hardness. J Biomater Appl.

[13] Santos GB, Medeiros IS, Fellows CE, Muench A, Braga RR (2007). Composite depth of cure obtained with QTH and LED units assessed by microhardness and micro-Raman spectroscopy. Oper Dent.

[14] Bennett AW, Watts DC (2004). Performance of two blue light-emitting-diode dental light curing units with distance and irradiation-time. Dent Mater.

[15] Lohbauer U, Rahiotis C, Krämer N, Petschelt A, Eliades G (2005). The effect of different light-curing units on fatigue behavior and degree of conversion of a resin composite. Dent Mater.

[16] Ceballos L, Fuentes MV, Tafalla H, Martínez A, Flores J, Rodríguez J (2009). Curing effectiveness of resin composites at different exposure times using LED and halogen units. Med Oral Patol Oral Cir Bucal.

[17] Mobarak E, Elsayad I, Ibrahim M, El-Badrawy W (2009). Effect of LED light-curing on the relative hardness of tooth-colored restorative materials. Oper Dent.

[18] Aguiar FH, Braceiro A, Lima DA, Ambrosano GM, Lovadino JR (2007). Effect of light curing modes and light curing time on the microhardness of a hybrid composite resin. J Contemp Dent Pract.

[19] Pilo R, Cardash HS (1992). Post-irradiation polymerization of different anterior and posterior visible light-activated resin composites. Dent Mater.

[20] Mutal L, Gani O (2005). Presence of pores and vacuoles in set endodontic sealers. Int Endod J.

[21] Moraes RR, Sinhoreti MA, Correr-Sobrinho L, Ogliari FA, Piva E, Petzhold CL (2010). Preparation and evaluation of dental resin luting agents with increasing content of bisphenol-A ethoxylated dimethacrylate. J Biomater Appl.

[22] Lee YK (2008). Influence of filler on the difference between the transmitted and reflected colors of experimental resin composites. Dent Mater.

[23] Antonson SA, Anusavice KJ (2001). Contrast ratio of veneering and core ceramics as a function of thickness. Int J Prosthodont.

[24] Santos MJ, Passos SP, da Encarnação MO, Santos GC, Bottino MA (2010). Hardening of a dual-cure resin cement using QTH and LED curing units. J Appl Oral Sci.

[25] Kilinc E, Antonson SA, Hardigan PC, Kesercioglu A (2011). The effect of ceramic restoration shade and thickness on the polymerization of light- and dual-cure resin cements. Oper Dent.

